# Nothing New under the Sun—“Needle Case”

**Published:** 1853-12

**Authors:** 


### NOTHING NEW UNDER THE SUN.

Very likely not: but here followeth a letter from a reputable and reliable physician, narrating a case, which, if not new, is at least something of an episode from the old paths. We withhold the full names and places, on account of the family interested, but can give them to any anxious enquirer, and can satisfy him of the authenticity of our source of information. The following extract is from a letter, in our possession, written by none else than the physician himself in attendance, and it offers some interesting *points.* We do not now suggest any explanation, but commend the nut to our readers, hoping they may crack it, each to his own taste.

"———— Aug. 16, 1853.

*Dear Sir:*

* * * * "The particulars of the rather interesting and somewhat perplexing 'needle-case' here, are substantially as follows: About the 1st of May, Miss Mary H., æt nineteen, accidentally and at different times plunged two needles into her arm, which I extracted. About a week afterwards she came to my office with her arm enormously swollen. I examined it without being able to discern the cause. She came the third time, May 22d, when four needles were detected at the upper edge of the swelling, very deep in the flesh, which I was obliged to cut, before getting them. 25th, four were taken. 29th, fourteen were found—some of these worked up to the shoulder. 30th, seventeen. 31st, took one from the arm and five from the left breast. I found needles from time to time till some time in July, amounting in all to 132—from a small cambric up to a large sized darning needle. Some of them were very rusty, and others quite black; some lay very deep under the muscles so that it was necessary to cut to the bone to get them, while others were so near the surface that but a slight incision was necessary.—Nearly all were taken from separate places. Thirty-five were found in the left breast, (if I remember correctly,) thirteen in the right breast, four in the left side, the rest in the left arm and shoulder.

Every suggestion that imagination and credulity could invent has been circulated, as to the cause of this really singular phenomenon. It is supposed by some, that after getting in the first, she become so excited that she put the rest of them in herself, in a state of somnambulism. She is a respectable young lady, of a respectable family, and affirms that she 'certainly knows nothing about it.' The family had a large box of needles whence she may have derived her supply.

After the first forty or fifty, it was evident from the appearance of the needles that they were placed there at intervals, a few at a time. The inflammation at one time was so great, it was feared she would lose her arm. It is well now, with the exception of being weak and somewhat crooked. These are the facts as far as I recollect. It would be a great satisfaction to know exactly how they came there.

In great haste, I remain yours, &c., C. S——"

---

EMPIRICISM.—We have always considered it in vain to raise a cry against the existence of quackery in the abstract, as long as the community prefer to support it, for, like pork or whiskey, it will be in market as long as there is a demand for it. While we regard quacks with unmingled censure and contempt, we both blame and pity those persons who are not only the chief supporters of such stupendous fraud and ignorance, but we are also its deepest victims.

A case in point lately occurred in New York City. A young woman sustained a life long injury in the use of a compound administered by a quack. The mother sued the proprietor of the mixture, and the Judge charged the jury, among other things, that "the same degree of skill ought not to be expected, and is not in law required, in cases like this, as where regular physicians are called in. Persons who take medicines from advertisements in newspapers, must, to a considerable extent, take the consequences."

